# Effectiveness of Inferior Vena Cava Filters without Anticoagulation Therapy for Prophylaxis of Recurrent Pulmonary Embolism

**DOI:** 10.5041/RMMJ.10246

**Published:** 2016-07-28

**Authors:** Miri Zektser, Carmi Bartal, Lior Zeller, Roman Nevzorov, Alan Jotkowitz, Vered Stavi, Vitaly Romanyuk, Gregory Chudakov, Leonid Barski

**Affiliations:** 1 Department of Internal Medicine F, Soroka University Medical Center, Beer-Sheva, Israel; 2 Department of Internal Medicine E, Soroka University Medical Center, Beer-Sheva, Israel; 3 Department of Radiology, Soroka University Medical Center, Beer-Sheva, Israel

**Keywords:** Deep vein thrombosis, inferior vena cava filter, pulmonary embolism

## Abstract

**Objective:**

The optimal treatment of deep vein thrombosis (DVT) is anticoagulation therapy. Inferior vena cava filter (IVC) placement is another option for the prevention of pulmonary embolism (PE) in patients with deep vein thrombosis. This is used mostly in patients with a contraindication to anticoagulant therapy. The purpose of the present study was to compare the two options.

**Methods:**

A retrospective cohort study of two groups of patients with DVT: patients who received an IVC filter and did not receive anticoagulation due to contraindications; and patients with DVT and similar burden of comorbidity treated with anticoagulation without IVC insertion. To adjust for a potential misbalance in baseline characteristics between the two groups, we performed matching for age, gender, and Charlson’s index, which is used to compute the burden of comorbid conditions. The primary outcome was an occurrence of a PE.

**Results:**

We studied 1,742 patients hospitalized with the diagnosis of DVT in our hospital;93 patients from this population received IVC filters. Charlson’s score index was significantly higher in the IVC filter group compared with the anticoagulation group. After matching of the groups of patients according to Charlson’s score index there were no significant differences in primary outcomes.

**Conclusion:**

Inferior vena cava filter without anticoagulation may be an alternative option for prevention of PE in patients with contraindications to anticoagulant therapy.

## INTRODUCTION

The most effective treatment for patients with deep vein thrombosis (DVT) is anticoagulation therapy. Traditionally, anticoagulant therapy involves parenteral anticoagulants (heparin, low-molecular heaprins), overlapping with and followed by oral vitamin K antagonists. Recently, new or direct oral anticoagulants (NOACs or DOACs), including the factor Xa inhibitors rivaroxaban, apixaban, and edoxaban, and the direct thrombin inhibitor dabigatran etexilate have been developed. These drugs have been approved for the treatment of acute deep vein thrombosis (DVT) and pulmonary embolism (PE).^1^–^5^

Another therapeutic option for DVT treatment is inferior vena cava (IVC) filter placement. The only widely accepted and validated indications for IVC filter placement are: (1) absolute contraindication to therapeutic anticoagulation; (2) failure of anticoagulation when there is acute proximal venous thrombosis; and (3) life-threatening hemorrhage on anticoagulation. There are other situations in which the indication for IVC filter placement is controversial.^6^

In the last decade there has been increased use of filters for treatment of DVT, including their use, in addition to anticoagulant therapy, in patients with PE and/or a large clot burden, poor cardiopulmonary reserve, or a suspected increased risk for recurrence.^7^–^9^ Their use has also been advocated by several guidelines.^10^–^12^ The results of the most recent investigations do not support such a strategy, demonstrating that, compared with anticoagulation alone, placement of a retrievable IVC filter for 3 months in addition to anticoagulation provided no benefit in terms of PE recurrence or mortality in patients presenting with an acute symptomatic PE.^13^

A number of percutaneous IVC filters have been developed since the introduction of the Greenfield filter in 1973. The newer devices are designed to optimize flow dynamics, maximize clot-trapping capacity, and increase ease of insertion.^6^,^14^–^16^ The newest devices are potentially retrievable. Despite the obvious theoretical benefit, carefully controlled trials that demonstrate the ability of IVC filters to decrease recurrence rates or mortality from PE have not been performed.^6^,^14^,^17^ While the data suggest that recurrent embolism is unusual following filter insertion (2%–4% in most series), strong scientific evidence that IVC filters prevent death from PE is not currently available.^18^

A systematic review of retrievable IVC filters estimated an incidence of PE following filter placement of 1.3%.^19^

An impact on case fatality rate with vena cava filters was shown in unstable patients, whether or not they received thrombolytic therapy, and in stable patients who received thrombolytic therapy. Vena cava filters were associated with a lower all-cause in-hospital case fatality rate compared with anticoagulation therapy only without IVC filters among unstable patients who received thrombolytic therapy (7.6% versus 18%) and lower all-cause case fatality rate in unstable patients who did not receive thrombolytic therapy (33% versus 51%).^7^

Previous studies suggest that the addition of IVC filter placement to standard anticoagulation results in a reduction in the risk of subsequent PE; however, filter placement increases the incidence of DVT. Filter placement does not appear to increase the overall risk of venous thromboembolism (VTE), post-thrombotic syndrome, or mortality.^20^,^21^

Of note, neither these studies nor any others have compared the efficacy of anticoagulation with IVC filter placement in the absence of anticoagulation.

Whether concomitant anticoagulant therapy should be utilized following filter placement is unknown. Although filter placement protects the pulmonary vascular bed, it does nothing to lessen the thrombotic predisposition or to decrease the incidence of lower-extremity venous thrombosis. Small thrombi are capable of passing through patent filters or through collaterals around obstructed filters; furthermore, direct thrombus extension can occur through the filter itself. Because patients with IVC filters are at risk for IVC thrombosis, insertion site thrombosis, and recurrence of the initial thromboembolic event, continued use of anticoagulants when there are no contraindications is advisable.^14^,^22^

The purpose of our study was to investigate the natural history of patients with DVT and IVC filter insertion without anticoagulation therapy and compare them with patients on anticoagulation only without IVC filter.

## METHODS

We performed a retrospective cohort study of patients with DVT admitted to the Soroka University Medical Center, a 1,000-bed tertiary care teaching hospital that serves as the only tertiary referral hospital for southern Israel (estimated population 1,000,000) between January 1, 2006 and January 1, 2010.

Two groups of patients with DVT were compared: patients who received an IVC filter and no anticoagulation; and patients with DVT and similar burden of comorbidity treated with anticoagulation without IVC insertion.

All patients in the IVC filter group were treated with IVC filter insertion and did not receive any anticoagulant drugs. In patients in the IVC filter group the contraindications for anticoagulant therapy were active bleeding, recent surgery, pre-existing life-threatening bleeding, various coagulopathies, and the inability to receive anticoagulant therapy per the decision of the senior attending physician.

To adjust for a potential misbalance in baseline characteristics between the group of patients who received anticoagulation and the patients that were implanted with an IVC filter, we performed matching for age, gender, and Charlson’s index, which is used to compute the burden of comorbid conditions. Charlson’s index is a list of 19 conditions each of which has a weight assigned from 1 to 6, derived from relative risk estimates of a proportional hazard regression model. The higher the score, the more severe the burden of comorbidity is, and the likelyhood of mortality increases.^23^ Charlson’s index is a well-validated indicator of overall disease burden and can be accurately calculated based on ICD-9 diagnoses.

The primary outcome was an occurrence of a PE. The secondary outcomes were 1-year all-cause mortality, 2-year all-cause mortality, a recurrent hospitalization for a thrombotic event in the first year after DVT diagnoses, and length of hospital stay.

The discharge diagnoses (ICD-9) were used to identify the subjects with DVT.

Deep vein thrombosis was defined as a positive compression ultrasonography study of the lower extremities. Pulmonary embolism was defined as the obstruction of the pulmonary artery or one of its branches due to thrombus revealed either on angiographic computed tomography study or ventilation-perfusion scan.

Information on the patients’ demographic characteristics, ICD-9 diagnoses, medications, and clinical and laboratory data was obtained during a comprehensive medical chart review and from the computerized hospital database.

The study was approved by the Institutional Review Board prior to its initiation.

The following types of IVC filters were used in the present study: Simon Nitinol Filter (SNF) (Bard Peripheral Vascular, Inc., Temple, AZ, USA), a non-retrievable filter, was inserted to 41 patients of this study; OptEase Filter (Cordis Endovascular; a Johnson & Johnson Company, Warren, NJ, USA), a retrievable filter, was inserted to 37 patients; Cook Celect Filter (William Cook Europe, Bjaeverskov, Denmark), a retrievable filter, was inserted to 11 patients; and ALN Filter (ALN Implants Chirurgicaux, Ghisonaccia, France), a retrievable filter, was inserted to 5 patients. All filters were placed and positioned by ultrasound guide with femoral or jugular insertion sites. No complications were demonstrated in filter placement.

### Statistical Analysis

The results are presented as the mean ± standard deviation for continuous variables and as number and percentage of total patients for categorical data. Student’s *t* test was used for comparison of the continuous variables and chi-square test for categorical data with the use of Fisher’s exact test if needed. We used the Mann–Whitney test for the comparison of continuous variables not distributed normally and presented as median and interquartile range (IQR). Survival curves were calculated by the Kaplan–Meier method, and comparison between groups of patients was performed by log-rank test. For multi-variable analysis, the binary logistic regression model was applied. The initial selection of the variables entered into the model was based on univariate analysis significance with an inclusion criterion of *P*<0.10. The results of multivariate analysis were presented as the hazard ratio (HR) with 95% confidence interval (CI). A two-sided *P* value <0.05 was considered statistically significant. The statistical analysis was performed with SPSS software (version 17.0).

## RESULTS

From January 2006 to December 2010 at Soroka University Medical Center there were 1,742 patients with DVT, and 93 patients from this population received IVC filters. General characteristics of the entire cohort are shown in Table 1. No age differences were found between the two groups of patients. The majority of the patients in the anticoagulation group were women (58.8% compared to 48.4% in IVC filter group, *P*=0.048).

**Table 1:** General Patient Characteristics.

Characteristics	IVC Filter Group *n*=93	Anticoagulation Group *n*=1649	*P* Value
Age (y), mean±SD	65.2±17.2	62.7±22.6	0.2
Female gender, *n* (%)	45 (48.4)	969 (58.8)	0.048
History of myocardial infarction, *n* (%)	14 (15.1)	328 (19.9)	0.3
Chronic heart failure, *n* (%)	6 (6.5)	211 (12.8)	0.07
Peripheral vascular disease, *n* (%)	9 (9.7)	301 (18.3)	0.035
Dementia, *n* (%)	11 (11.8)	214 (13.0)	0.7
Chronic obstructive lung disease, *n* (%)	4 (4.3)	170 (10.3)	0.06
Connective tissue disease, *n* (%)	5 (5.4)	52 (3.2)	0.2
History of stroke, *n* (%)	19 (20.4)	218 (13.2)	0.048
Liver cirrhosis, *n* (%)	2 (2.2)	48 (2.9)	0.7
Chronic renal failure, *n* (%)	34 (36.6)	559 (33.9)	0.6
Diabetes mellitus, *n* (%)	23 (24.7)	453 (27.5)	0.6
Solid tumor, *n* (%)	41 (44.1)	403 (24.4)	<0.001
Leukemia, *n* (%)	1 (1.1)	13 (0.8)	0.5
Lymphoma, *n* (%)	4 (4.3)	44 (2.7)	0.3
Metastatic tumor, *n* (%)	26 (28.0)	189 (11.5)	<0.001
Charlson score, median (IQR)	4 (2; 8)	3 (0; 6)	<0.001

IQR, interquartile range (25th; 75th percentiles).

Patients in the IVC filter group compared with the anticoagulation group had more chronic medical conditions prior to hospitalization, including peripheral vascular disease, cerebral vascular disease, hemiplegia, and solid and metastatic tumors. Therefore, Charlson’s score index was significantly higher in patients in the IVC filter group compared with the anticoagulation group [4 (IQR 2; 8) versus 3 (IQR 0; 6), *P*<0.001].

Clinical outcomes before matching are shown in Table 2. The rate of 1-year and 2-year mortality before matching according to severe comorbid diseases was significantly higher in patients in the IVC filter group compared to the anticoagulation group (49.5% versus 24.3%, *P*<0.001; 52.7% versus 30.5%, *P*<0.001, respectively). No significant difference was found between the two groups of patients for 1-year readmission.

**Table 2:** Outcomes.

Outcomes	IVC Filter Group *n*=93	Anticoagulation Group *n*=1649	*P* Value
1-Year readmission, *n* (%)	83 (89.5)	1402 (85.0)	0.3
1-Year mortality, *n* (%)	46 (49.5)	400 (24.3)	<0.001
2-Year mortality, *n* (%)	49 (52.7)	503 (30.5)	<0.001

Multivariate analysis of factors associated with 1-year mortality (Table 3) revealed that advanced age and metastatic tumor were independent predictors (HR 1.01, 95% CI 1.006–1.01; HR 6.1, 95% CI 4.99–7.42, respectively).

**Table 3:** Multivariate Analysis for 1-Year Mortality.

Variables	Hazard Ratio	95% CI	*P* Value
Age (the increment for each year)	1.01	1.006–1.01	<0.001
Metastatic tumor	6.1	4.99–7.42	<0.001

**Figure 1:**
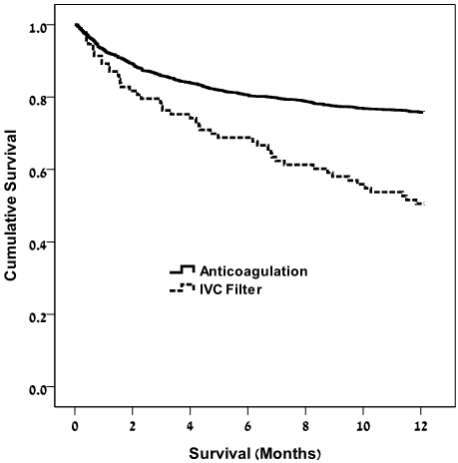
Kaplan–Meier Survival Plots for 1-year Survival. Stratified by IVC filter and anticoagulation. Log-rank test *P*<0.001.

Figure 1 shows the Kaplan–Meier survival plots for 1-year survival stratified by IVC filter and anticoagulation and demonstrates a decreased survival of patients in the IVC filter group compared to the anticoagulation group.

After matching both groups of patients according to Charlson’s score index there were no significant differences in primary (occurrence of PE) or secondary outcomes (1-year readmission, 1-year mortality, 2-year mortality) (Table 4).

**Table 4:** Outcomes after Matching.

Outcomes	IVC Filter Group *n*=92	Anticoagulation Group *n*=92	*P* Value
Pulmonary embolism, *n* (%)	6 (6.5)	3 (3.2)	0.5

1-Year readmission, *n* (%)	82 (89.1)	79 (85.9)	0.6

1-Year mortality, *n* (%)	45 (48.9)	32 (34.8)	0.1
2-Year mortality, *n* (%)	48 (52.2)	39 (42.4)	0.2

## DISSCUSSION

In the present study IVC filters were used for the prophylaxis of recurrent PE in patients with DVT. The insertion of an IVC filter was a treatment option only for those patients who were diagnosed recently with a DVT, with contraindications to anticoagulant therapy. This is the only strict indication for IVC insertion published in guidelines of the American College of Chest Physicians,^24^ American Heart Association,^12^ and the British Committee for Standards in Hematology.^22^ There is no consensus on the role of IVC filters in reducing mortality or recurrent PE in patients with other indications, such as patients with VTE despite anticoagulation, patients with recent VTE requiring anticoagulation while awaiting surgery, or primary prevention in high-risk patients. This could be a possible explanation for the observed outcomes in our patients: after performing matching of the two groups of patients we found no significant differences in primary and secondary outcomes between the two groups. This confirms the utility of using IVC filters in patients with absolute contraindications to anticoagulation. It is possible that if, in our institution, the IVC was inserted for other indications the results would be less favorable in the filter group. The non-selective use of IVC filters is associated with unacceptable morbidity and mortality, and only a few patients among those surveyed would have benefited from an IVC filter.^25^

The mortality rate in our study was high, even before matching the group of patients with DVT without IVC filter insertion. In fact, the majority of patients in both groups in this study hospitalized with a diagnosis of DVT had several severe comorbidities, including malignant metastatic tumors, cerebral vascular disease with hemiplegia, myocardial infarction, congestive heart failure, chronic lung diseases, chronic liver diseases, and chronic kidney diseases; they also had a high Charlson’s score index. It is possible that other less complicated patients with diagnoses of DVT may be treated as outpatients with good results.^7^,^19^,^26^

In the IVC filter group about 50% of the patients had cancer, and 28% had a metastatic tumor; the percentage was significantly higher than in the no-IVC filter group. Cancer itself, or its treatment, may result in certain clinical complications that make systemic anticoagulation very risky. Many recent studies questioned the need to insert IVC filters in cancer patients, particularly in those with advanced-stage disease whose survival is short and in whom prevention of PE may be of little clinical benefit and could be a poor utilization of resources.^27^,^28^ Although in our study we did not perform formal subgroup analysis, the IVC filter group, which had a higher proportion of cancer patients, did not have a higher 1- or 2-year mortality compared to the other group.

Although more than 50% of our patients were inserted with retrievable filters, none of the filters was removed during our 2 years of observation. In this group of patients almost 50% died in the first 2 years, and as was mentioned previously this group of patients had significant comorbidities. No randomized clinical trials have been performed to compare retrievable and non-retrievable filters. In one large retrospective study of retrievable filters placed for various indications, only 18.7% of the filters were successfully retrieved.^29^

Significant complications after IVC filter insertion were not demonstrated in this study. Our study did not reveal an increased incidence of hospitalization due to recurrent thrombotic events, and this might allay the fears of those clinicians who might be reluctant to place IVC filters due to the concern of the filter being a nexus for thrombosis.

Only 10 patients in the group of filter insertion received anticoagulation during 2 years of follow-up. This demonstrates that the contraindications for anticoagulation in the majority of patients were not temporary, but rather persistent.

In addition, significant complications after IVC filter insertion were not demonstrated in this study.

Not surprisingly, multivariate analysis for 1-year mortality confirms that advanced age and metastatic tumor were predictors of 1-year mortality in the present study.

The present study adds to the growing body of literature that an effective treatment for patients with DVT and an absolute contraindication to anticoagulation is an IVC filter.

## LIMITATIONS OF THIS STUDY

This was a retrospective study performed in only one medical center. The patients on anticoagulation in our study did not receive a NOAC.

## CONCLUSION

Inferior vena cava filter without anticoagulation may be an alternative therapeutic option for prevention of PE in patients with contraindications to anticoagulant therapy.

## Abbreviations

DOAC: direct oral anticoagulant
DVT: deep vein thrombosis
IVC: inferior vena cava
NOAC: new oral anticoagulant
PE: pulmonary embolism
VTE: venous thromboembolism

## References

[1] Franco P, Losub DI (2015). New anticoagulants in the management of venous thromboembolism in women. Thromb Res.

[2] Arcelus JI, Domènech P, Fernández-Capitan Mdel C (2015). Rivaroxaban in the treatment of venous thromboembolism and the prevention of recurrences: a practical approach. Clin Appl Thromb Hemost.

[3] Yeh CH, Hogg K, Weitz JI (2015). Overview of the new oral anticoagulants: opportunities and challenges. Arterioscler Thromb Vasc Biol.

[4] Sharifi M, Freeman W, Bay C, Sharifi M, Schwartz F (2015). Low incidence of post-thrombotic syndrome in patients treated with new oral anticoagulants and percutaneous endovenous intervention for lower extremity deep venous thrombosis. Vasc Med.

[5] Bacchus F, Schulman S (2015). Clinical experience with the new oral anticoagulants for treatment of venous thromboembolism. Arterioscler Thromb Vasc Biol.

[6] Streiff MB (2000). Vena caval filters: a comprehensive review. Blood.

[7] Stein PD, Matta F, Keyes DC, Willyerd GL (2012). Impact of vena cava filters on in hospital case fatality rate from pulmonary embolism. Am J Med.

[8] Stein PD, Kayali F, Olson RE (2004). Twenty-one–year trends in the use of inferior vena cava filters. Arch Intern Med.

[9] Spencer FA, Bates SM, Goldberg RJ (2010). A population-based study of inferior vena cava filters in patients with acute venous thromboembolism. Arch Intern Med.

[10] Kaufman JA, Kinney TB, Streiff MB (2006). Guidelines for the use of retrievable and convertible vena cava filters: report from the Society of Interventional Radiology multidisciplinary consensus conference. J Vasc Interv Radiol.

[11] Caplin DM, Nikolic B, Kalva SP (2011). Quality improvement guidelines for the performance of inferior vena cava filter placement for the prevention of pulmonary embolism. J Vasc Interv Radiol.

[12] Jaff MR, McMurtry MS, Archer SL (2011). Management of massive and submassive pulmonary embolism, iliofemoral deep vein thrombosis, and chronic thromboembolic pulmonary hypertension: a scientific statement from the American Heart Association. Circulation.

[13] Mismetti P, Laporte S, Pellerin O, PREPIC2 Study Group (2015). Effect of a retrievable inferior vena cava filter plus anticoagulation vs anticoagulation alone on risk of recurrent pulmonary embolism: a randomized clinical trial. JAMA.

[14] Becker DM, Philbrick JT, Selby JB (1992). Inferior vena cava filters. Indications, safety, effectiveness. Arch Intern Med.

[15] Kinney TB (2003). Update on inferior vena cava filters. J Vasc Interv Radiol.

[16] Kercher K, Sing RF (2003). Overview of current inferior vena cava filters. Am Surg.

[17] Girard P, Stern JB, Parent F (2002). Medical literature and vena cava filters: so far so weak. Chest.

[18] Ku GH, Billett HH (2005). Long lives, short indications. The case for removable inferior cava filters. Thromb Haemost.

[19] Angel LF, Tapson V, Galgon RE, Restrepo MI, Kaufman J (2011). Systematic review of the use of retrievable inferior vena cava filters. J Vasc Interv Radiol.

[20] Decousus H, Leizorovicz A, Parent F (1998). A clinical trial of vena caval filters in the prevention of pulmonary embolism in patients with proximal deep-vein thrombosis. Prévention du Risque d’Embolie Pulmonaire par Interruption Cave Study Group. N Engl J Med.

[21] PREPIC Study Group (2005). Eight-year follow-up of patients with permanent vena cava filters in the prevention of pulmonary embolism: the PREPIC (Prevention du Risque d’Embolie Pulmonaire par Interruption Cave) randomized study. Circulation.

[22] Baglin TP, Brush J, Streiff M, British Committee for Standards in Haematology Writing Group (2006). Guidelines on use of vena cava filters. Br J Haematol.

[23] O’Connel RL, Lim LL (2000). Utility of the Charlson comorbidity index computed from routinely collected hospital discharge diagnosis codes. Methods Inf Med.

[24] Kearon C, Akl EA, Comerota AJ (2012). Antithrombotic therapy and prevention of thrombosis, 9th ed: American College of Chest Physicians evidence-based clinical practice guidelines. Chest.

[25] Thomsen MB, Lindblad B, Bergqvist D (1994). Fatal pulmonary embolism in an unselected series: the possible role of caval filters in prevention. Eur J Surg.

[26] Carson JL, Kelley MA, Duff A (1992). The clinical course of pulmonary embolism. N Engl J Med.

[27] Karabinis VD, Mehta V, Dupont EL, Matsumoto T, Kerstein MD (1993). Potential of overuse of the inferior vena cava filter. Surg Gynecol Obstet.

[28] Jarrett BP, Dougherty MJ, Calligaro KD (2002). Inferior vena cava filters in malignant disease. J Vasc Surg.

[29] Barginear MF, Lesser M, Akerman ML (2009). Need for inferior vena cava filters in cancer patients: a surrogate marker for poor outcome. Clin Appl Thromb Hemost.

